# A Novel Whole-Body Wearable Technology for Motor Assessment in Multiple Sclerosis: Feasibility and Usability Pilot Study

**DOI:** 10.3390/s25196214

**Published:** 2025-10-07

**Authors:** Jessica Podda, Erica Grange, Claudia Latella, Andrea Tacchino, Enrico Valli, Ludovica Danovaro, Gianluca Milani, Marco Forleo, Antonella Tatarelli, Davide Gorbani, Alex Coppola, Ludovico Pedullà, Giampaolo Brichetto, Daniele Pucci

**Affiliations:** 1 neuroBRITE Research Center, Italian Multiple Sclerosis Foundation, 16149 Genoa, Italy; erica.grange@fismets.it (E.G.); andrea.tacchino@aism.it (A.T.); ludovico.pedulla@aism.it (L.P.); giampaolo.brichetto@aism.it (G.B.); 2 Artificial and Mechanical Intelligence laboratory, Italian Institute of Technology, 16163 Genoa, Italy; claudia.latella@iit.it (C.L.); enrico.valli@iit.it (E.V.); ludovica.danovaro@iit.it (L.D.); gianluca.milani@iit.it (G.M.); marco.forleo@iit.it (M.F.); antonella.tatarelli@iit.it (A.T.); davide.gorbani@iit.it (D.G.); alex.coppola@iit.it (A.C.); daniele.pucci@iit.it (D.P.)

**Keywords:** wearable sensor, wearable technology, motor assessment, AI, multiple sclerosis, usability

## Abstract

(1) Background: Technological advancements provide new opportunities to objectively assess motor deficits in people with Multiple Sclerosis (PwMS). This pilot study aimed to evaluate the performance and usability of iFeel, a novel wearable system which integrates inertial sensors, instrumented shoes, and an AI-based algorithm. (2) Methods: Sixteen adult PwMS (Expanded Disability Status Scale—EDSS ≤ 6) performed motor tests (Timed 25-Foot Walk—T25FW; Timed Up and Go—TUG) both with and without the iFeel suit. Patient-reported outcomes (PROs) were also collected to assess perceived fatigue, dual-task impact, and walking difficulties. System Usability Scale (SUS) and ad hoc questionnaires have been further administered to test usability. (3) Results: No significant differences were found between the clinician and system-based scores for both T25FW (*p* = 0.383) and TUG (*p* = 0.447). Reliability analyses showed good agreement for T25FW (Intraclass Correlation Coefficient—ICC = 0.83) and excellent agreement for TUG (ICC = 0.92). Sensor-derived measures correlated strongly with PROs on fatigue, dual-task interference, and mobility. Usability was rated high (SUS: 78.6 ± 16.1), with participants reporting minimal discomfort and positive perceptions of iFeel usefulness for rehabilitation, health monitoring, and daily activities. (4) Conclusions: This pilot study provides preliminary yet promising evidence on the feasibility, usability, and perceived usefulness of the iFeel technology for motor assessment in PwMS. The findings support its further development and potential integration into clinical practice, particularly for remote or continuous motor monitoring.

## 1. Introduction

Multiple Sclerosis (MS) is the most common chronic inflammatory, demyelinating, and neurodegenerative disease of the central nervous system in young adults, characterized by a variety of clinical symptoms [1] that profoundly affect the quality of life of people with MS (PwMS). Among these symptoms, a loss of motor function is often the most visible sign in PwMS [2]. Since the characteristics of MS symptoms can rapidly fluctuate within the same day or across days, precise, reliable, and valid measures are essential for identifying who may benefit from early interventions to improve motor functioning. Validated walking tests, such as the Timed-25 Foot Walk Test (T25FW) [3] and the Timed-Up and Go Test (TUG) [4], are widely recommended as more objective measures of motor impairment. However, they often fail to detect subtle deficits. Subjective evaluations of gait and balance typically integrated with objective assessments, using self-rating questionnaires and clinician-assessed tests such as the 12-item Multiple Sclerosis Walking Scale (MSWS-12) [5] and Activity-Specific Balance Confidence (ABC) scale [6], respectively, also lack the sensitivity for the continuous recording of motor deficits. Given the limitations of conventional clinical measures, novel technologies may provide more sensitive, continuous, and valid assessments. Thus, the rising adoption of digital wearable technologies also represents promising tools for MS, providing more objective, less rater-dependent, and fine-grained functional outcomes with the potential for greater sensitivity to short-term changes than physical exam-based metrics [7]. Recently, several pieces of evidence demonstrated the potential of digital technologies that integrated gyroscopes and accelerometers applied with sophisticated AI-driven tools to evaluate and monitor motor functions both in laboratory and real-time settings [8,9]. By simultaneously capturing signals from different body segments, whole-body sensor systems offer a more detailed, precise, and holistic view of gait functions and postural control. Indeed, Tulipani and colleagues [10] demonstrated that sensor-derived acceleration metrics can predict fall risk in PwMS, highlighting the potential of wearable sensors to capture mobility-related deficits beyond standard clinical tests. Similarly, a validated multi-sensor system demonstrated excellent reliability compared with traditional clinical tests in different clinical populations including MS [11]. In addition, Chitnis and colleagues [12] found that outcomes collected by three sensors highly correlated with gold-standard clinical scales like the Expanded Disability Status Scale (EDSS), the Multiple Sclerosis Functional Composite, and the T25FW. Despite these advancements, the usability of such novel devices has rarely been systematically evaluated. Building on these considerations, the present pilot study has two main aims: (1) to evaluate the capabilities of sensor-based measures derived from a novel whole-body wearable perception system (iFeel), which comprises a network of wearable inertial devices, a pair of instrumented shoes with embedded force/torque sensors, and an AI-based estimation algorithm, by examining their correlation with traditional clinical outcome measures; (2) to assess the usability and user experience of the iFeel system in PwMS, with a specific focus on comfort, ease of use, acceptability, and privacy issues.

## 2. Materials and Methods

### 2.1. Participants

PwMS who were outpatients at the Italian MS Society (AISM) Rehabilitation Service in Genoa (Italy) have been enrolled. Inclusion criteria were an age of 18 years and older, a confirmed MS diagnosis following the McDonald criteria [13], all disease courses (relapsing remitting—RRMS, secondary progressive—SPMS, and primary progressive—PPMS), the absence of relapsing in the last 3 months, an EDSS ≤ 6, and adequate visual and hearing capabilities. Exclusion criteria were a Montreal Cognitive Assessment (MoCA) [14] score < 24, neurological and major psychiatric illness, a history of learning disability, serious head trauma (causing coma), and alcohol or drug abuse. The study was approved by the Local Ethics Committee (CER Liguria 319/2022) and conducted in accordance with the ethical standards of the Declaration of Helsinki as revised in 2013. All participants provided written informed consent to participate in the study. Data collection took place between January 2022 and November 2024.

### 2.2. The Whole-Body Technology: iFeel

The iFeel system is an advanced full-body wearable perception platform developed by the Artificial and Mechanical Intelligence (AMI) Laboratory at the Italian Institute of Technology (Genoa, Italy). It comprises

- A distributed network of wearable sensing devices;
- A pair of sensorized shoes equipped with force/torque sensors;
- An AI-driven algorithm for real-time estimation.

This integrated system enables the real-time tracking of human kinematics (e.g., position, velocity, and acceleration) and the estimation of dynamics (e.g., external/internal forces and joint torques). The modular design of the wearable electronics ensures scalability in terms of both the number of wearable units and the types of sensors that can be integrated. Furthermore, iFeel can be deployed in diverse environments, including clinical settings, rehabilitation centers, and research laboratories, making it a valuable tool for monitoring people with neurological conditions such as MS.

The core of the iFeel hardware is the *node*, a compact unit capable of acquiring real-time motion and biometric data and transmitting it to a central receiving station. This station acts as a network controller and forwards the data to a host computer via a high-throughput USB serial communication protocol.

**Figure 1:**
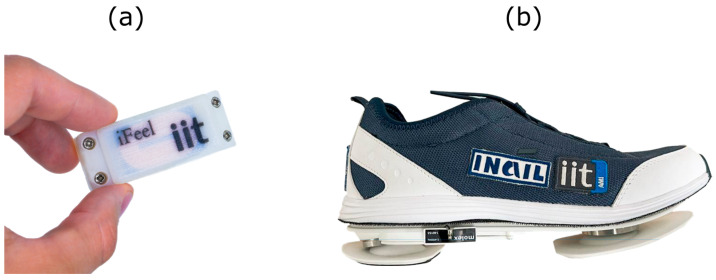
The iFeel components. (**a**) Wearable nodes; (**b**) sensorized shoes for ground force detection.

Each node (Figure 1a) includes

- A microcontroller-based electronics core with integrated power management and radio communication;
- An Inertial Measurement Unit (IMU) that provides acceleration data at 1 kHz and fused motion data at 100 Hz;
- A medical-grade infrared (IR) temperature sensor for high-resolution skin temperature monitoring;
- A haptic feedback transducer to simulate tactile sensations and enhance user interaction.

Additionally, iFeel includes a pair of sensorized shoes (Figure 1b), each equipped with four force/torque sensors to measure ground reaction forces during locomotion.

**Figure 2:**
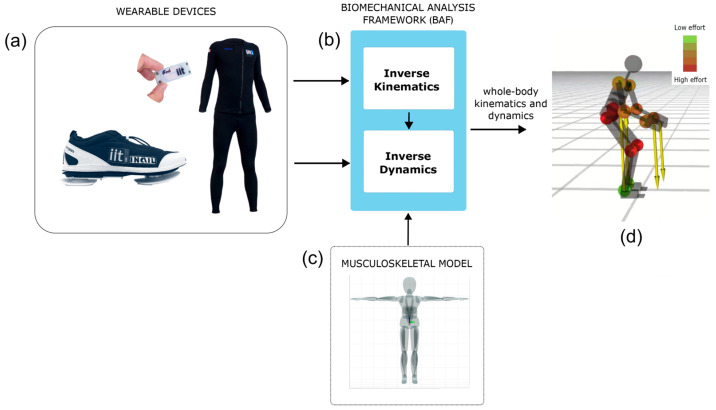
The iFeel software architecture. (**a**) Wearable iFeel devices including nodes and sensorized shoes. (**b**) Biomechanical Analysis Framework (BAF). (**c**) Muskoloskeletal model of the user. (**d**) Output: Whole-body human kinematics and dynamics.

The iFeel software architecture is built upon the Biomechanical Analysis Framework (BAF), a modular C++ framework designed to manage the entire processing pipeline. Both iFeel and BAF are research prototype implementations currently available as repositories on GitHub. The present study used the latest commit as of November 2024. As shown in Figure 2a, the system starts with the acquisition of multimodal human data through distributed wearable sensors. In the subsequent stage (Figure 2b), the BAF software architecture organizes and processes these inputs to enable real-time biomechanical analysis. A dedicated kinematics estimation module (Figure 2c) then fuses IMU data with a user-specific musculoskeletal model using constrained inverse kinematics algorithms, ensuring joint configurations with respect to physiological limits. Finally, the framework provides the real-time estimation of whole-body biomechanics—including joint positions, velocities, accelerations, external and internal forces, and joint torques (Figure 2d). Data streams from all nodes are synchronized at 60 Hz. Each acquisition cycle (≈16 ms) is fully processed by the estimation algorithm in less than 15 ms, including sensor fusion, constrained inverse kinematics, and dynamics estimation. This guarantees that each frame is reconstructed in real-time before the arrival of the next, without backlog or frame loss. All kinematic and dynamic variables are also stored for offline post-processing and KPI extraction. During the dynamic estimation stage, kinematic quantities are combined with force/torque measurements to estimate joint torques, internal wrenches, and external forces acting on the body. The formulation relies on a Stack-of-Tasks Maximum A Posteriori (SoT-MAP) estimator, which integrates sensor measurements, model constraints, and prior knowledge, enabling even the estimation of external forces in the absence of direct sensors. The framework supports both the real-time visualization of biomechanical variables and the offline analysis of recorded data, ensuring flexibility for clinical, research, and rehabilitation applications.

**Figure 3:**
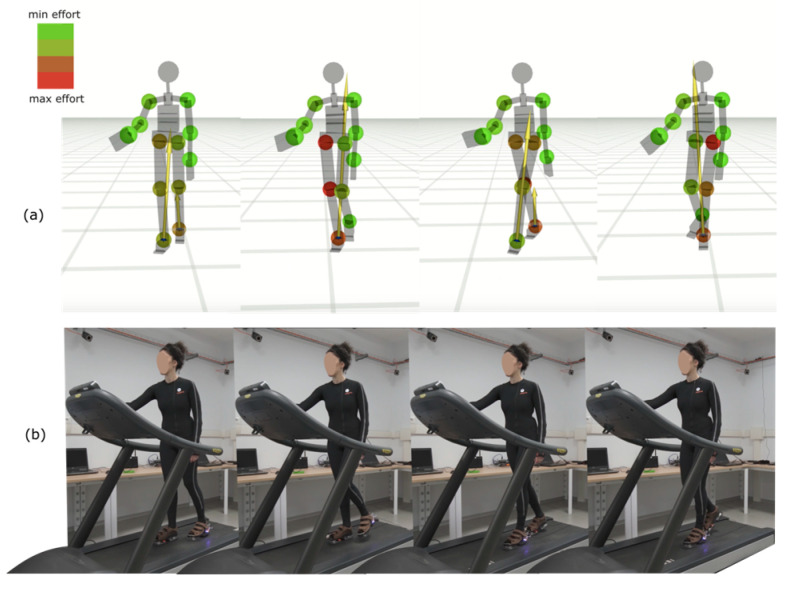
Visualization of joint torques and treadmill walking with the iFeel system. (**a**) Spheres represent joint torques, with colors ranging from green (low effort) to red (high effort). (**b**) Subject walking on a treadmill equipped with the iFeel wearable system. Please note that treadmill testing was performed exclusively during technical validation in the AMI laboratory, whereas all patient assessments were conducted using standard floor-based protocols (T25FW and TUG) at the AISM Rehabilitation Service.

The framework has been validated through human gait analysis on a treadmill. Real-time visualization includes joint markers represented as color-coded spheres, where the color indicates joint effort (green for minimal effort, red for maximal effort) (Figure 3). The system also supports offline data processing, allowing users to analyze previously recorded data. Thanks to BAF flexibility, the real-time C++ algorithms can be wrapped for use in MATLAB R2021B, facilitating post-processing and further analysis.

### 2.3. Procedure

After giving their informed written consent to participate in the study, demographic (age, educational level, gender) and disease-related (MS disease course, disease duration, EDSS, medications) information was collected from each participant. PwMS have been asked to perform traditional motor clinical tests, T25FW and TUG, without (traditional assessment) and with iFeel technology (sensorized assessment). PwMS also fulfilled the following patient reported outcomes (PROs) to assess

- The impact of MS on an individual’s walking ability (MSWS-12) [5];
- The individual perception on dual-task impact on common daily activities (Daily living Activities Questionnaire—DIDA-Q), divided into cognition (DIDA-Q_COG) and balance and mobility (DIDA-Q_BAL & MOB) [15];
- The perceived fatigue in terms of physical (MFIS_P), cognitive (MFIS_C), and psychosocial functioning (MFIS_PS) (Modified Fatigue Impact Scale—MFIS) [16].

Both the T25FW and the TUG tests were performed twice for each participant. After interacting with iFeel, participants evaluated the technology components in terms of effectiveness, efficiency, and satisfaction using the System Usability Scale (SUS) [17] and other ad hoc usability questionnaires. Before wearing the iFeel suit, the PwMS completed a questionnaire assessing their previous experience with technologies and related emotions. These measures were based on modified versions of the validated questionnaire by Venkatesh and colleagues (2012) [18]. Following the experimental session, participants were asked to evaluate the perceived usefulness and ease of use of the iFeel suit (adapted from [18,19]), along with its aesthetics and ergonomic comfort (adapted from [20]) and privacy and security (adapted from [21]). The duration of the entire experimental session was approximately 90 min.

### 2.4. Key Performance Indicators (KPsI)

**Figure 4:**
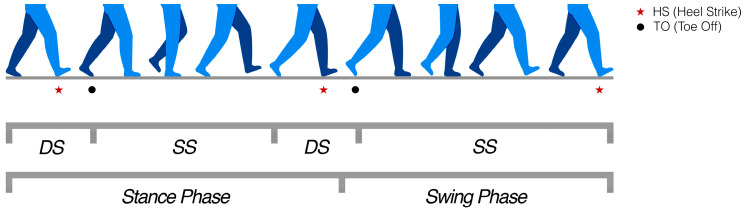
Representation of the right leg gait cycle phases with heel strike (HS), toe off (TO), stance–swing, and single–double support periods.

To create a subject-specific model for each participant, anthropometric data were collected, including height, weight, dominance, shoe size, pelvis depth, shoulder width and height, torso depth, neck, arm, forearm, thigh, and leg diameters, as well as hand height and width. The gait analysis relies on data acquired from the iFeel sensorized shoes. The vertical component of the ground reaction forces (GRFs), measured by force–torque sensors embedded in the shoes, is used to identify the main gait events: heel strike (HS), when the foot first contacts the ground, and toe off (TO), when the foot completely leaves the ground. These two events define the boundaries of the gait cycle, defined as the interval between two consecutive HSs of the same foot. Each cycle can be subdivided into the stance phase (foot in contact with the ground), the swing phase (foot moving in the air), the single support phase (SS), during which only one foot is in contact with the ground, and periods of double support (DS), when both feet simultaneously touch the ground. Figure 4 provides a graphical representation of the main gait events (HS and TO) for the right leg, along with the subdivision of the gait cycle into stance–swing and single–double support phases.

Once HS and TO events are extracted for both feet, the algorithm isolates the steady-state walking portion, discarding gait initiation and termination, during which variability is usually higher. This ensures that the KPIs are computed only from consistent and repetitive cycles. Based on the study by [16], the following Key Performance Indicators (KPIs) were extracted:

- Cycle duration (s): the elapsed time between two consecutive HSs of the same foot.
- Cadence (steps/min): a measure of walking rhythm, representing how quickly a person takes steps. It is derived from the total number of heel strikes during steady walking and expressed as steps per minute.
- Stride length (m): the distance covered between two consecutive HSs of the same foot, estimated from the foot trajectory reconstructed via kinematic data.
- Walking speed (m/s): the average forward velocity, obtained by dividing stride length by cycle duration.
- Double support (% of cycle): the percentage of the gait cycle during which both feet are simultaneously in contact with the ground. This corresponds to the sum of the initial and terminal double support phases.
- Stance phase (% of cycle): the proportion of the gait cycle in which a foot is in contact with the ground, from HS until the subsequent TO of the same foot.
- Swing phase (% of cycle): the complementary portion of the cycle, from TO until the following HS of the same foot, when the foot moves through the air to prepare for the next step.
- Maximum angular velocity (deg/s): the peak angular velocity of the foot during swing, obtained from the inertial and kinematic measurements of the shoes.
- Swing width (% of stride length): the medio-lateral deviation of the foot trajectory during swing, normalized by stride length, providing an indicator of gait stability.
- Path length (% of stride length): the actual length of the trajectory traced by the foot during swing, expressed relative to stride length. Values above 100% indicate less direct and more irregular trajectories.

These KPIs together capture the essential temporal, spatial, and dynamic features of locomotion. In this study, they were used to evaluate gait and balance in PwMS, where even small deviations in timing or trajectory can reveal compensatory strategies or impairments.

### 2.5. Statistical Analysis

Descriptive statistics were calculated for demographic and clinical variables. Specifically, continuous variables were summarized by their mean (M), standard deviation (SD), or standard error (SE); categorical variables were described as counts and percentages. We assessed the normal distribution of the measurements using a Shapiro–Wilk test with a significance level set at a = 0.05.

Paired *t*-tests were conducted to compare performances between traditional clinical tests and their sensorized versions using iFeel technology. Only the first trial has been considered to capture raw and unbiased participants’ performance. First, we investigated any difference between scores from the first trial, expressed in terms of meter or time, calculated by the clinician and automatically by the technology (i.e., clinician vs. iFeel) during the iFeel assessment. Second, we compared the clinician-derived score for the traditional condition (without iFeel) with that obtained for the sensorized condition (with iFeel). The absolute agreement between the traditional and sensorized measurements was tested using the Intraclass Correlation Coefficient (ICC): with a two-way random effects model, absolute agreement, 95% confidence intervals (95% CI). Values lower than 0.5, between 0.5 and 0.75, between 0.75 and 0.9, and larger than 0.90 indicated poor, moderate, good, and excellent agreement, respectively [22]. Pearson’s correlations were run to assess the associations between clinical motor tests and the perceived impact of MS on individual’s walking ability, and the dual-task impact on common daily activities and fatigue, as measured by MSWS-12, DIDA-Q, and MFIS, respectively. Correlation coefficients (r) ranging from 0.20 to 0.39 were considered moderate, from 0.40 to 0.59 as relatively strong, from 0.60 to 0.79 as strong, and higher as a very strong correlation [23].

Known groups analysis was used to evaluate whether an instrument can discriminate between known groups of PwMS expected to score differently on the measure of interest. Groups were defined using the MSWS-12 cut-off value of 25 [24], discriminating between PwMS with none-to-minimal (MSWS-12 < 25) or mild-to-moderate (MSWS-12 ≥ 25) perceived walking limitations. For study purposes, we focused the additional KPI analyses on the T25FW, given its simple linear path and suitability for evaluating the discriminative potential of the iFeel system. A Multivariate Analysis of Variance (MANOVA) was then conducted with KPIs as dependent variables. Participants were categorized into known groups based on their perceived walking difficulties, and the side affected (left or right) was included as a fixed factor to account for potential lateralization effects. All confidence intervals were set as two-sided (95% confidence levels). Effect sizes were calculated using partial eta squared (partial η^2^). Conventional benchmarks classify values of 0.01, 0.06, and 0.14 as small, medium, and large effects, respectively [25]. Usability questionnaires were analyzed using M and SD. Statistical analyses were performed using SPSS Software (Version 23) for Windows.

## 3. Results

### 3.1. Sample Demographic and Clinical Characteristics

A total of 16 PwMS (9 female) completed the study. Their age was 55.2 years (SD = 8.0) with a moderate EDSS as indicated by the score of 3.3 (SD = 1.3). See Table 1 for a detailed description of the PwMS’ demographic and clinical characteristics.

### 3.2. Assessing the Agreement Between Clinician and Machine with iFeel and Exploring the Effect of Sensorization on Test Performance

Paired *t*-tests were conducted to compare performances between traditional clinical tests and their sensorized versions using iFeel technology. First, we compared scores obtained during the iFeel assessments when computed manually by the clinician versus automatically by the system. Paired *t*-tests did not reveal statistically significant differences in performance for both T25FW (*p* = 0.383) and TUG (*p* = 0.447), suggesting a comparable performance across methods (see Table 2).

Second, we compared the clinician-derived performance scores between the traditional and sensorized conditions. Paired *t*-tests revealed significant differences (all *p* ≤ 0.001), showing that PwMS tended to be slower during the sensorized assessments than in those computed with traditional ones (see Table 3).

The ICC analysis showed high agreement between the modalities. Specifically, the ICC values indicated good agreement for the T25FW (0.83) and excellent agreement for the TUG (0.92), suggesting that, despite differences in absolute performance, the sensorized and traditional versions consistently ranked individuals’ performance.

**Figure 5:**
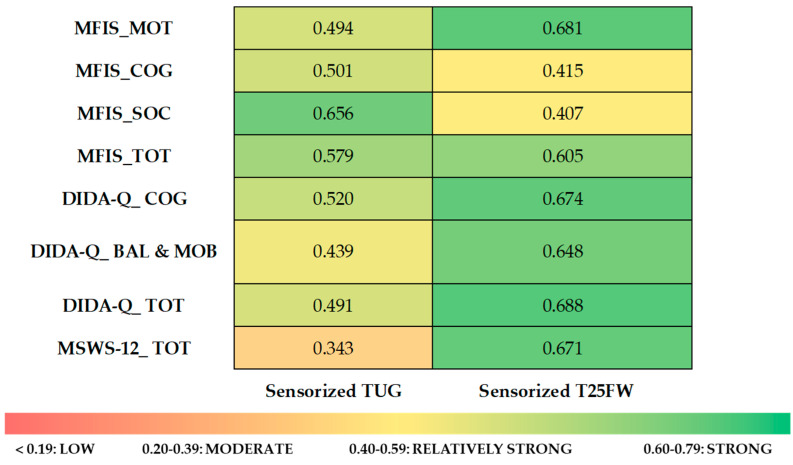
Heat map of correlation analyses between the sensorized TUG and T25FW and PROs. The chart visually represents the strength of correlations, ranging from red (low) to green (high). Exact correlation coefficients are provided in Table 4.

Pearson’s correlations revealed associations between iFeel-derived scores and PROs. Sensorized T25FW scores showed strong correlations with the physical and total scores of the MFIS, all subscales and total scores of the DIDA-Q, and the MSWS-12 (all *p* < 0.01). Sensorized TUG scores were moderately to strongly correlated with the cognitive and psychosocial subscales of the MFIS, its total score, and the cognitive subscale of the DIDA-Q (all *p* < 0.05). Exact correlation coefficients are reported in Table 4, while Figure 5 presents a heat map illustrating the strength of these correlations.

The MANOVA results indicated a significant main effect of MSWS-12 subgroups, suggesting that PwMS with no or low perceived walking difficulties (MSWS-12 < 25; N = 8) and those with moderate to high perceived difficulties (MSWS-12 ≥ 25; N = 8) differed significantly in cycle duration (left and right), stance phase, left swing phase, and max angular velocity left (*p* = 0.010; *p* = 0.002; *p* = 0.020; *p* = 0.020, and *p* = 0.034, respectively) (see Table 5). However, no other significant main effects were found for the side affected (left: 8 N; right: 8 N; all ps > 0.05), and no interaction was observed between MSWS-12 subgroups and the side affected (all ps > 0.05).

### 3.3. iFeel Technology Usability

The average SUS score was 78.6 ± 16.1, which is well above the cut-off of 68, indicating that the iFeel Technology exhibits good usability [17]. A summary of the key usability domains investigated is presented below.

*Experience with innovation and technology, and related emotions.* Before testing iFeel, PwMS reported very low levels of fear (M = 4.4 ± 0.8) and discomfort (M = 1.7 ± 0.9) associated with using technology. On average, they expressed a good level of curiosity (M = 3.6 ± 1.1), liking (M = 3.7 ± 1.2), and willingness to attend training courses on how to use technology (M = 3.7 ± 1.4). Participants reported feeling not very nervous (M = 1.7 ± 0.8) or insecure (M = 2.6 ± 1.1) and perceiving themselves as somewhat capable (M = 3.2 ± 1.1), with limited advanced technological skills (M = 2.7 ± 1.0), and moderate ease of use (M = 3.0 ± 1.0). These findings suggest that PwMS were open and motivated to engage with innovative technologies, which is encouraging for future home-based or remote monitoring applications.

*Perceived usefulness and ease of use of the technology.* After interacting with iFeel, participants reported that the system could significantly improve their health condition (M = 3.9 ± 0.8), support rehabilitation intervention (M = 4.0 ± 0.8), and enhance overall quality of life (M = 3.8 ± 0.7). Participants found iFeel useful for monitoring their health at home (M = 3.9 ± 0.8), easy to use (M = 4.1 ± 0.6), and not requiring significant effort (M = 3.9 ± 0.7). They expressed their willingness to use it in the future at their home (M = 3.7 ± 1.1), and at work to protect their own health (M = 3.5 ± 1.1). These results highlighted the potential of iFeel for real-world adoption and self-managed rehabilitation, complementing standard clinical care.

*Aesthetics and ergonomic comfort.* PwMS reported that the suit was aesthetically pleasant (M = 3.5 ± 0.9), had a good style (M = 3.4 ± 1.2), and was comfortably tailored (M = 3.5 ± 1.0). They did not feel awkward while wearing it (M = 1.8 ± 0.8), and the suit allowed them to move freely (M = 3.8 ± 1.0). Overall, they considered the iFeel suit to be highly adequate for performing the task (M = 3.8 ± 0.9). These ergonomic and aesthetic qualities are crucial to ensure adherence in repeated or long-term use scenarios.

*Privacy and trust.* PwMS believed that the health-related data were collected reliably (M = 4.6 ± 0.6) and could be handled confidentially (M = 4.6 ± 0.7). They did not express concerns about the confidentiality of the data collected (M = 4.1 ± 1.5), nor did they think that the use of this technology could harm their health (M = 1 ± 0) or that transferring data to a social robot would pose a health risk (M = 1.1 ± 0.3). This topic is particularly important for enabling remote monitoring and integrating iFeel into routine clinical and home-based care.

## 4. Discussion

This pilot study explored the potential of a novel whole-body wearable system (iFeel), comprising a network of wearable inertial devices, a pair of instrumented shoes, and an AI-based estimation algorithm, by examining their correlation with traditional clinical motor measures in MS. Overall, the results showed that the measures collected through iFeel can capture motor functions in a sample of PwMS and strongly correlated with traditional tests. Interestingly, the measures collected by the clinician and the iFeel system were highly comparable for the T25FW and TUG, suggesting that both modalities provide similar accuracy in assessing task duration. The instrumented shoes may have introduced subtle modifications to natural gait patterns, potentially influencing the accuracy of the recorded metrics, as indicated by the statistical significance found while comparing scores computed by the clinicians across all tests considered. However, the good to strong ICCs across clinical assessments (all ICCs > 0.75) indicated that the performance ranking among participants remained stable: individuals who were faster or walked more in the traditional tests tended to maintain their relative position in the sensor-based versions as well. Although the iFeel-based tests resulted in slightly longer completion times, the measurements proved to be reliable and comparable with the traditional format. This suggests that the sensorized assessment did not compromise the validity of the test; rather, it preserved the ability to distinguish individual differences in performance, which is essential for both clinical and research applications. These results indicate that iFeel measures could serve as clinically relevant indicators, supporting their integration into rehabilitation and patient monitoring. Additionally, to explore iFeel’s discriminative potential, KPI-based analyses were restricted to the T25FW, due to its simple and linear path, which allowed for the more controlled interpretation of gait-related metrics. In our study, PwMS were categorized into two subgroups according to the MSWS-12 benchmarks (none to minimal perceived walking limitations: 0 ≤ MSWS-12 < 25; mild to moderate perceived walking limitations: 25 ≤ MSWS-12 < 75). Several KPIs successfully distinguished PwMS reporting no or low perceived walking difficulties and those with moderate to high perceived difficulties. Specifically, cycle duration for both the left and right foot in the T25FW differentiated the two subgroups, suggesting that, beyond total execution time, fine-grained spatio-temporal variables can further capture subtle motor differences among participants. This result aligns with [26], which showed that mean gait parameters measured with inertial sensors are suitable for separating PwMS with different disability levels. Spatio-temporal variables, such as walk duration and cadence, serve as fundamental indicators of gait efficiency and overall mobility. In line with Carpinella and colleagues [24], these spatio-temporal variables could be sensitive biomarkers for detecting subtle impairments, revealing asymmetries that may not be apparent through simple observation. This, in turn, can support more accurate diagnoses and the development of tailored rehabilitation programs. Furthermore, our results proved that a sensorized assessment of gait was associated with perceived walking ability in PwMS. The lack of significant effects for the affected side, as indicated by the non-significant MANOVA results, suggests that the observed group differences are robust and not simply attributable to lateralized motor impairment. This finding reinforces the validity of spatio-temporal metrics in capturing perceived walking difficulties, regardless of whether the left or right side is affected.

The significant correlations observed between sensor-derived T25FW and TUG and PROs, including fatigue (MFIS), perceived cognitive difficulties (DIDA-Q), and walking ability (MSWS-12), suggest that these objective metrics may reflect aspects of functional impairment that are meaningful to patients’ daily lives. This aligns with previous studies that reported associations between perceived fatigue and walking difficulties with sensor-derived measures from the T25FW, suggesting that integrating subjective and objective domains provides superior predictive power compared with using either in isolation, addressing a critical gap in understanding the factors influencing the activity levels of PwMS [27]. Notably, these significant correlations support a multimodal assessment framework in MS, in which technological tools complement, rather than replace, self-reported experiences, highlighting the role of wearable and sensor-based technologies in providing sensitive, real-time measures of disability that extend beyond clinic-based evaluations [28,29].

Usability is particularly important when wearable devices are intended for use during real-world tasks or activities, such as walking, which is often considered a vital sign, especially in chronic conditions [30]. If walking is a key activity being measured by wearables, it is important to understand how usable these devices are in this context [31]. The results of this study provide encouraging evidence regarding the usability and user acceptance of the iFeel Technology among PwMS. The SUS score of 78.6, well above the standard cut-off of 68, suggests that the system was perceived as highly usable, even by individuals who may not consider themselves technologically proficient. This finding is particularly relevant, as the adoption of wearable technologies in rehabilitation depends not only on clinical effectiveness but also on user engagement and sustained use. Following the testing phase, participants rated the system as highly useful for supporting rehabilitation, improving quality of life, and enabling health monitoring at home. These perceptions are critical in the context of chronic neurological conditions like MS, where continuity of care and autonomy in disease management are essential. Furthermore, the aesthetic and ergonomic features of the suit, including comfort, style, and freedom of movement, contributed to a positive user experience, reinforcing the importance of considering both functional and psychosocial factors in the design of wearable technologies. Overall, these findings suggest that iFeel Technology is not only functionally usable but also psychologically acceptable and socially appropriate for PwMS, aligning with the view that, for wearable devices to be widely accepted, they must be easy to wear, easy to use, affordable, feature relevant functionality, and be aesthetically pleasing [31]. Failure to assess usability may result in the development of devices that are not worn or that are worn or used incorrectly and thus may negatively impact data collection and quality while also reducing adherence [32]. These positive perceptions suggest that the technology could be readily adopted in home-based or occupational settings, supporting self-managed rehabilitation and continuous monitoring. Conversely, any discomfort, awkwardness, or perceived difficulty could limit adherence and reduce the effectiveness of remote or long-term interventions. Therefore, ergonomic design, aesthetic appeal, and user-friendly interaction are essential not only for usability testing but also for ensuring meaningful engagement in real-world scenarios. Future studies should explore long-term adherence and effectiveness in larger and more diverse cohorts and investigate how the personalization of the technology can further enhance user engagement.

Finally, although this study is the first to demonstrate the potential of a novel whole-body sensor technology for assessing gait in PwMS, the relatively small sample size limits the generalizability of the findings. As a pilot investigation, our primary objective was to establish feasibility and usability, rather than to draw definitive clinical conclusions. Nevertheless, future studies should include a larger and more diverse cohort to increase statistical power, enhance the robustness of the results, and allow for subgroup analyses based on disease severity, clinical phenotype, or assistive device use. To enhance the generalizability and interpretability of the results, future studies should include a control group of healthy participants, allowing for direct comparisons and a clearer distinction between pathological and physiological gait features, which is particularly relevant when assessing compensatory strategies or subtle motor impairments. Such work would also enable longitudinal follow-up studies to evaluate the sensitivity of iFeel technology in detecting changes over time, monitoring disease progression, and assessing the effects of rehabilitation or pharmacological interventions.

This study represents the first application of the iFeel Technology in a clinical population. Beyond confirming feasibility and acceptance in PwMS, this pilot also provided valuable insights for the iterative refinement of the system. The real-world deployment made it possible to identify specific technical limitations and data-related issues that may have influenced data completeness and the interpretation of gait metrics. The most relevant were

- IMU data loss due to occasional 2.45 GHz wireless communication dropouts. Across all sessions, packet loss remained limited (<20% of the acquired stream at 60 Hz) and did not compromise the computation of gait cycles, as sufficient steady-state data were available for KPI extraction. Moreover, the pipeline was designed to log synchronized data across nodes, allowing corrupted packets to be identified and excluded. Ongoing developments focus on the implementation of a fully wired sensing suit with centralized Wi-Fi connectivity, which is expected to substantially minimize packet loss by eliminating interference from the 2.45 GHz channel. In parallel, post-processing gap-filling algorithms are being developed to reconstruct short missing segments (time windows <200 ms) through interpolation and model-based estimation, thereby maintaining the continuity of biomechanical signals.
- Modeling inaccuracies in human link dimensions within the biomechanical model, which may affect joint trajectory reconstruction. This issue is being addressed by the integration of an enhanced URDF-based anthropometric model.
- Sensor displacement relative to anatomical landmarks during data acquisition, occasionally reducing accuracy. This limitation underscores the importance of improved ergonomics and fixation supports, which are currently under refinement.
- Inverse kinematics resolution, which may reduce the detail in reconstructed joint trajectories, though they are still adequate for detecting clinically meaningful differences.

From a computational perspective, iFeel is capable of processing complete biomechanical estimation cycles in <15 ms, matching the 60 Hz acquisition rate. This ensures that outputs are generated continuously in real time without perceptible delays, thereby meeting the temporal requirements of clinical gait assessments. Another relevant methodological aspect is the rationale for adopting a constrained inverse kinematics (IK) framework instead of machine learning (ML) approaches. IK provides interpretable and biomechanically grounded outputs such as joint angles and torques, computed within a model that respects anatomical limits. This makes the results readily understandable to clinicians and directly linked to physiology. ML techniques (e.g., neural networks, SVMs) are promising but raise issues of generalization and transparency. PwMS often show atypical gait patterns that may fall outside training distributions, leading to reduced robustness. By contrast, IK remains valid across subjects provided that the anthropometric model is personalized.

With respect to IMU drift, we acknowledge that IK does not inherently provide drift correction. However, in our protocol this limitation is practically mitigated by performing rapid re-calibration procedures at the end of each task, ensuring stable orientation estimates without the need for complex or non-transparent filtering methods. Finally, in terms of computational performance, our IK pipeline completes kinematic and dynamic estimation within 15 ms per frame at 60 Hz acquisition, offering deterministic real-time operation on standard hardware and meeting the temporal requirements of clinical gait assessments. Although ML inference can be fast, its efficiency depends heavily on model complexity and hardware availability, and remains less predictable in clinical contexts. For these reasons, IK represents a conservative but appropriate starting point, combining interpretability, robustness, and predictable performance. ML remains part of our roadmap, especially as a complementary tool (e.g., sensor drift correction or multimodal fusion) once larger heterogeneous datasets are available.

Concerning sensor integration, the iFeel system adopts a feature-level fusion strategy. IMUs already provide orientation estimates by combining accelerometer, gyroscope, and magnetometer data through embedded fusion algorithms, while the instrumented shoes deliver calibrated force/torque values from strain gauges after a dedicated calibration. Using these processed features enables the reconstruction of full-body kinematics and dynamics in a modular, efficient, and real-time manner, without re-implementing low-level filters and with reduced computational overhead.

Alternative schemes were considered but are not appropriate in this context. Early/data-level fusion, which combines all raw signals, would increase complexity and noise, with little advantage over the calibrated features already available. Late/decision fusion is more suitable for discrete classification tasks (e.g., fall detection) but cannot provide the continuous kinematic and dynamic estimation required here. Thus, feature-level fusion represents the best compromise between efficiency, modularity, and clinical relevance. Future work may explore hybrid ML-based fusion, but for continuous gait assessment in PwMS, feature-level fusion is the most appropriate choice. Overall, these observations show that the current version of iFeel is already suitable for feasibility and usability studies in PwMS, but further improvements are still needed before large-scale clinical use. The planned hardware and software upgrades, such as more reliable communication, better anthropometric modeling, strategies to handle data loss, and ML-based IMU drift correction, should reduce the current limitations. These steps are expected to make the system more accurate and robust, supporting its future use for the continuous and sensitive monitoring of motor function in everyday clinical practice. In addition, the observed slowdown when using instrumented shoes may be interpreted in two ways. On one hand, it could reflect an ecological usability challenge, as participants had to adapt to additional weight and possible constraints imposed by the device. On the other hand, it might represent a methodological artifact related to participants’ increased caution when performing tasks with unfamiliar equipment. Future studies will incorporate familiarization sessions and continue to optimize device design to minimize potential usability barriers.

## 5. Conclusions

This pilot study provides preliminary yet promising evidence regarding the feasibility, usability, and perceived usefulness of the iFeel technology for motor assessment in PwMS. Despite some limitations related to sample size and technical challenges, the results suggest for the first time that this wearable sensor system is well accepted by PwMS and capable of capturing relevant gait-related data. These findings have clear clinical implications. iFeel reliably captures motor function and correlates with PROs, allowing clinicians to monitor progress and detect subtle changes. The high agreement with traditional assessments supports its use alongside standard evaluations, while its remote capabilities offer practical opportunities for tele-rehabilitation and ongoing patient monitoring, particularly for individuals with mobility limitations. Importantly, this initial deployment in a clinical setting not only confirmed the potential of the technology, but also offered critical insights to guide its refinement. As the technology continues to evolve, future studies with a larger sample and improved system robustness will be essential to validate its clinical applicability and support its integration into routine neurorehabilitation pathways for PwMS.

## Figures and Tables

**Table 1:** Sample demographic and clinical characteristics.

Characteristic		Value
Age (in years), mean (SD)		55.2 (8.0)
Years of education, mean (SD)		13.2 (3.5)
Disease duration, mean (SD)		12.6 (8.7)
MS course, n (%)	RRMS	12 (75%)
	SPMS	3 (19%)
	PPMS	1 (6%)
EDSS, mean (SD) score		3.3 (1.3)
Medications, n (%)	Siponimod	2 (12.5%)
	Gilenya	2 (12.5%)
	Tecfidera	1 (6%)
	Aubagio	1 (6%)
	Natalizumab	1 (6%)
	Copaxone	1 (6%)
	Ocrelizumab	1 (6%)
	Avonex	1 (6%)
	No treatment	6 (38%)
MFIS_P, mean (SD) score		15.7 (9.5)
MFIS_C, mean (SD) score		11.7 (7.9)
MFIS_PS, mean (SD) score		2.2 (1.8)
MFIS_TOT, mean (SD) score		29.7 (17.0)
MSWS-12, mean (SD) score		27.7 (11.6)
DIDA-Q_COG, mean (SD) score		5.9 (5.4)
DIDA-Q_BAL & MOB, mean (SD) score		12.7 (2.2)
DIDA-Q_TOT, mean (SD) score		18.6 (13.6)

EDSS: Expanded Disability Status Scale; MFIS_P: physical subscale of the Modified Fatigue Impact Scale; MFIS_C: cognitive subscale of the Modified Fatigue Impact Scale; MFIS_PS: psychosocial subscale of the Modified Fatigue Impact Scale; MFIS_TOT: total score of the Modified Fatigue Impact Scale; MSWS-12: 12 item-Multiple Sclerosis Walking Scale; DIDA-Q_COG: cognition subscale of the Daily living Activities Questionnaire; DIDA-Q_BAL & MOB: balance and mobility subscale of the Daily living Activities Questionnaire; DIDA-Q_TOT: total score of the Daily living Activities Questionnaire.

**Table 2:** Paired *t*-test results comparing clinician vs. system-computed scores during iFeel assessment.

Test	N	M	SE	*p*	M Diff	Mean SE	95% CI	
							Min	Max
Clinician-scored T25FW (s)	16	8.2	0.5	0.383	−0.238	0.265	−0.80	0.32
System-scored T25FW (s)	16	8.4	0.5					
Clinician-scored TUG (s)	16	9.9	0.5	0.447	−0.284	0.364	−1.06	0.49
System-scored TUG (s)	16	10.26	0.6					

**Table 3:** Paired *t*-test results from traditional and sensorized assessment (scores computed by the clinician).

Test	N	M	SE	*p*	M Diff	Mean SE	95% CI	
							Min	Max
Traditional T25FW	16	6.9	0.3	0.001	−1.339	0.298	−1.97	−0.70
Sensorized T25FW	16	8.2	0.5					
Traditional TUG	16	9.9	0.5	<0.001	−2.33	0.343	−3.06	−1.60
Sensorized TUG	16	12.2	0.8					

**Table 4:** Bivariate Pearson’s correlation results between sensorized clinical tests and PROs.

		MFIS_MOT	MFIS_COG	MFIS_SOC	MFIS_TOT	DIDA-Q_COG	DIDA-Q_BAL & MOB	DIDA-Q_TOT	MSWS-12_TOT
Sensorized T25FW	r	0.681	0.415	0.407	0.605	0.674	0.648	0.688	0.671
	***p***	**0.004**	0.110	0.118	**0.013**	**0.004**	**0.007**	**0.003**	**0.004**
Sensorized TUG	r	0.494	0.501	0.656	0.579	0.520	0.439	0.491	0.343
	***p***	0.052	**0.048**	**0.006**	**0.019**	**0.039**	0.089	0.053	0.194

Bold values represent significant differences.

**Table 5:** Results from MANOVA for T25FW on spatio-temporal KPIs.

KPI	Subgroup	M	SE	95% CI		*p*	Partial η^2^
				Min	Max		
cadence (steps/min)	MSWS < 25	99.733	4.073	90.859	108.607	0.99	0.210
	MSWS ≥ 25	89.433	4.073	80.559	98.307		
cycle_duration_left (s)	MSWS < 25	1.126	0.045	1.029	1.223	**0.01**	0.441
	MSWS ≥ 25	1.320	0.045	1.223	1.417		
cycle_duration_right (s)	MSWS < 25	1.130	0.037	1.049	1.211	**0.002**	0.570
	MSWS ≥ 25	1.339	0.037	1.258	1.419		
stride_length_left (m)	MSWS < 25	0.730	0.052	0.616	0.843	0.223	0.121
	MSWS ≥ 25	0.635	0.052	0.522	0.749		
stride_length_right (m)	MSWS < 25	0.754	0.066	0.610	0.897	0.635	0.019
	MSWS ≥ 25	0.708	0.066	0.565	0.852		
max_angular_velocity_left (deg/s)	MSWS < 25	2.064	0.193	1.643	2.485	0.648	0.018
	MSWS ≥ 25	1.936	0.193	1.516	2.357		
max_angular_velocity_right (deg/s)	MSWS < 25	2.569	0.163	2.214	2.923	**0.034**	0.321
	MSWS ≥ 25	2.020	0.163	1.666	2.375		
swing_width_left (% of stride length)	MSWS < 25	32.473	8.991	12.883	52.063	0.404	0.059
	MSWS ≥ 25	43.477	8.991	23.887	63.067		
swing_width_right (% of stride length)	MSWS < 25	44.793	6.754	30.077	59.510	0.542	0.032
	MSWS ≥ 25	38.798	6.754	24.081	53.515		
path_length_left (% of stride length)	MSWS < 25	111.450	11.931	85.454	137.446	0.632	0.020
	MSWS ≥ 25	119.737	11.931	93.741	145.733		
path_length_right (% of stride length)	MSWS < 25	126.712	11.134	102.452	150.971	0.405	0.058
	MSWS ≥ 25	113.119	11.134	88.859	137.378		
stance_phase_left (% of cycle)	MSWS < 25	62.007	0.659	60.571	63.443	**0.020**	0.377
	MSWS ≥ 25	64.517	0.659	63.082	65.953		
stance_phase_right (% of cycle)	MSWS < 25	64.123	1.211	61.484	66.763	0.281	0.096
	MSWS ≥ 25	66.058	1.211	63.418	68.697		
swing_phase_left (% of cycle)	MSWS < 25	37.993	0.659	36.557	39.429	**0.020**	0.377
	MSWS ≥ 25	35.483	0.659	34.047	36.918		
swing_phase_right (% of cycle)	MSWS < 25	35.877	1.211	33.237	38.516	0.281	0.096
	MSWS ≥ 25	33.942	1.211	31.303	36.582		

Bold values represent significant differences.

## Data Availability

Data will be available on demand from the corresponding author. The data are not publicly available due to privacy reasons.

## Abbreviations

The following abbreviations are used in this manuscript:

DIDA-Q_BAL & MOB	Balance and mobility subscale of the Daily living Activities Questionnaire
DIDA-Q_COG	Cognition subscale of the Daily living Activities Questionnaire
DIDA-Q_TOT	Total score of the Daily living Activities Questionnaire
EDSS	Expanded Disability Status Scale
ICC	Intraclass correlation coefficient
M	Mean
MFIS_C	Cognitive subscale of the Modified Fatigue Impact Scale
MFIS_P	Physical subscale of the Modified Fatigue Impact Scale
MFIS_PS	Psychosocial subscale of the Modified Fatigue Impact Scale
MFIS_TOT	Total score of the Modified Fatigue Impact Scale
MS	Multiple Sclerosis
MSWS-12	12-item Multiple Sclerosis Walking Scale
PPMS	Primary progressive Multiple Sclerosis
PwMS	People with Multiple Sclerosis
RRMS	Relapsing–remitting Multiple Sclerosis
SD	Standard deviation
SE	Standard error
SPMS	Secondary progressive Multiple Sclerosis
SUS	System Usability Scale
T25FW	Timed-25 Foot Walk Test
TUG	Timed-Up and Go Test
